# Higher Reactive Oxygen Species and cellular aging in oral mucosal cells of young smokers: a comparative analytical study

**DOI:** 10.3389/froh.2025.1372494

**Published:** 2025-02-14

**Authors:** Bushra Imdad, Uzair Abbas, Pershad Kumar, Durre Sameen Kamran, Mahtab Khan, Niaz Hussain, Muhib Ullah Khalid

**Affiliations:** ^1^ Department of Physiology, Dow University of Health Sciences, Karachi, Pakistan; ^2^ Department of Pulmonology, Jinnah Postgraduate Medical Center, Karachi, Pakistan; ^3^ Department of Pathology, Dow University of Health Sciences, Karachi, Pakistan; ^4^ Dow University of Health Sciences, Karachi, Pakistan; ^5^ Liaquat University of Medical and Health Sciences, Jamshoro, Pakistan

**Keywords:** smoking, Reactive Oxygen Species, cellular aging, young smokers, oral mucosal cells

## Abstract

**Background:**

Cigarette smoke contains chemical components that cause excessive production of Reactive Oxygen Species (ROS), altering cell physiology and affecting key pathways. This leads to hyperinflammation, DNA damage, and cellular aging, which may cause oral and pulmonary pathologies. Our study aims to investigate the impact of smoking on ROS levels and cellular aging in oral mucosal cells. We compared Reactive oxygen Species and cellular aging between smokers and non-smokers. Secondarily, we also compared the results between young and old smokers.

**Methods:**

Oral swabs were taken from 50 smokers and 50 nonsmokers using a cytology brush. We quantified the reactive oxygen species (ROS) by using oxidized 2'7' dichlorodihydrofluorescein-diacetate (DCFH-DA) dye. To assess cellular aging, mRNA levels of the CYR61 gene-a cellular aging marker, were compared through RT-PCR.

**Results:**

It was found that smokers had a higher percentage of ROS in comparison to non-smokers (*p* value < 0.001). Additionally, there was an over-expression of the CYR61 gene in smokers as compared to non-smokers (*p* value = 0.001). Furthermore, when comparing ROS and cellular aging between young smokers and old smokers, it was noted that there was a significantly higher percentage of ROS and up-regulation of mRNA levels of CYR61 gene in young smokers in comparison to old smokers (*p* value 0.001 and <0.0001 respectively).

**Conclusion:**

It has been observed that smokers have a higher amount of ROS production and cellular aging in their oral mucosal cells. In young smokers, ROS and cellular aging were found to be higher compared to older smokers. This is quite concerning and could be a major factor leading to oral pathologies in smokers.

## Introduction

According to the World Health Organization (WHO), cigarette smoking is 22.2% prevalent worldwide and Pakistan has 34% prevalence (1). Cigarette smoke (CS) adversely affect every vital organ of the body by altering several key cellular pathways and has become one of the leading cause of early death (2).

Cigarette smoking is a combustion process that leads to the aerosolization of thousands of toxic chemicals including carbon monoxide, hydrogen cyanide, and polycyclic aromatic hydrocarbons (3). Many of the components in CS chemically react with oxygen to generate free radicals and inhibit protective antioxidants (4). Through the combustion of noxious chemicals and generation of harmful reactive oxygen species (ROS), CS induces widespread tissue damage in a manner that mimics biological aging (5). Many *in vitro* and *in vivo* studies have elucidated mechanisms involved in cigarette smoke–induced inflammation, DNA damage, and autophagy, and the subsequent cell fates, including cell death, cellular senescence, and transformation mentioning key cause of over production of ROS (6, 7).

Physiologically, ROS molecules are formed by the partial reduction of molecular oxygen (O_2_) and it reacts with other molecules and play a role in cell development, proliferation, differentiation, oxygen sensing and immunity through reversible oxidative modifications of macromolecules; but when present in excess, they can cause cellular oxidative damage (8). CS induces overproduction of ROS molecules which leads to oxidation of macro molecules and cause their conversion into peroxynitrite, hydroquinone and hydrogen peroxide which in turn cause genomic instability, oxidative DNA damage, shortening of telomere length, protein oxidation and lipid oxidation in lung epithelial and other supporting cells (9).

Prior studies have shown that tobacco smoking induces premature cellular senescence in lung epithelial cells and basal progenitor cells (10, 11). CS directly and via over production of ROS induces early cellular aging in lung tissues and nasal epithelial cells (12). Radical- and oxidant-mediated modification of proteins, nucleic acids, lipids, sugars, and consequent damage of cells play crucial roles in the genesis of a large number of age-related diseases (13).

The first effected organs from smoking are oral and nasal cavities. There are multiple studies available of impact of ROS due to CS on nasal cavity, bronchial and pulmonary tissues but limited data is available on its impact on oral cavity in smokers. This study was designed to see the impact of CS on ROS production and cellular aging in oral mucosal cells of smokers in comparison with non-smokers. We also evaluated the differential ROS and cellular aging between young and old smokers.

## Methods

### Study design and settings

This cross-sectional study was performed from November 2022 to July 2023. The study was performed at the Department of Oral Health Sciences, Dow University of Health Sciences. Participants were recruited after written informed consent.

### Study participants

We recruited 100 study participants of either gender from age 18 or above. They were further divided into 50 smokers and 50 non-smokers (who never smoked). We also divided smokers into young smokers (age = 18–35 years) and old smokers (60 years and above) as per classification of WHO.

### Inclusion and exclusion criteria

Participants without any diagnosed oral pathological lesion (Lichen planus Leukoplakia, Aphthous stomatitis, Macule etc.) and with no other addiction were included. Whereas individuals with any other addiction habit (i.e., Gutka, alcohol), metabolic and inflammatory diseases, history of cardiovascular symptoms were excluded from the study.

### Sample size calculation

Sample size was calculated from NCSS PASS software with 95% CI and 80% power of the test. The calculated sample size was 100.

### Sample collection

We collected 100 oral mucosal cells through exfoliated oral cytology method with cytology brush with the help of dental technician after written informed consent from the participants. Samples were stored in Roswell Park Memorial Institute (RPMI) media in −80C for further use.

#### Intracellular ROS quantification

The ROS were quantified by using oxidized 2'7' Dichloro-dihydro-fluorescein-diacetate (DCFDA) staining as per manufacturer's instructions. Final concentration of 10 µM of dye was used and each sample was run in duplicate. Half of each sample was unstained and used as negative control while Hydrogen peroxide (H2O2) was used as positive control.

2′,7′-dichlorofluorescein (DCF) was measured by flow cytometry (FACS Canto II). Data was analyzed in BD FACSDiva™ Software (Supplementary Figures 1 and 2).

#### Extraction of RNA and cDNA synthesis to quantify mRNA expression of CYR61 gene

RNA was extracted from oral mucosal cells by using Trizol™ reagent, according to manufactures protocol. After extraction, 500 ng RNA was used to synthesis cDNA. The reaction mix was prepared by using Thermo Scientific Revert Aid First Strand cDNA Synthesis Kit with cyclic condition as denaturation at 95℃ for 5 min, Annealing at 42℃ for 60 min, and elongation at 70℃ for 7 min. The synthesized cDNA was used for the RT-PCR for the quantitation of mRNA levels of CYR61 gene with 2X Maxima SYBR Green according to the manufacture's protocol. Comparative values were expressed as 2^−ΔΔCT^ with b-actin as housekeeping gene using following primers.

CYR61 Gene:

F: 5′-GAGTGGGTCTGTGACGAGGAT-3′

R: 5′-GGTTGTATAGGATGCGAGGCT-3′

Beta Actin Gene:

F: 5′-GATCATTGCTCCTCCTGAGC-3′

R: 5′-ACTCCTGCTTGCTGATCCAC-3′

### Statistical analysis

Data was entered in excel and then transferred to SPSS version 22.0. Mean of age and percentage of gender was calculated. Student's T-test was performed to compare the mean ROS and CYR61 gene expression between smokers and non-smokers. Using a 95% confidence interval (CI), a *p* value of less than 0.05 was considered as significant.

## Results

### Demographic profile of participants

The mean age of smokers and non-smokers was 41.29 ± 9.12 and 39.14 ± 8.34 years respectively. There were 80% male in smokers and 90% males in non-smokers groups. The mean age of young smokers was 28.45 ± 4.56 years and of old smokers was 58.18 ± 8.76 years respectively (*p* = 0.003).

### Comparison of ROS between smokers and non-smokers

**Figure 1:**
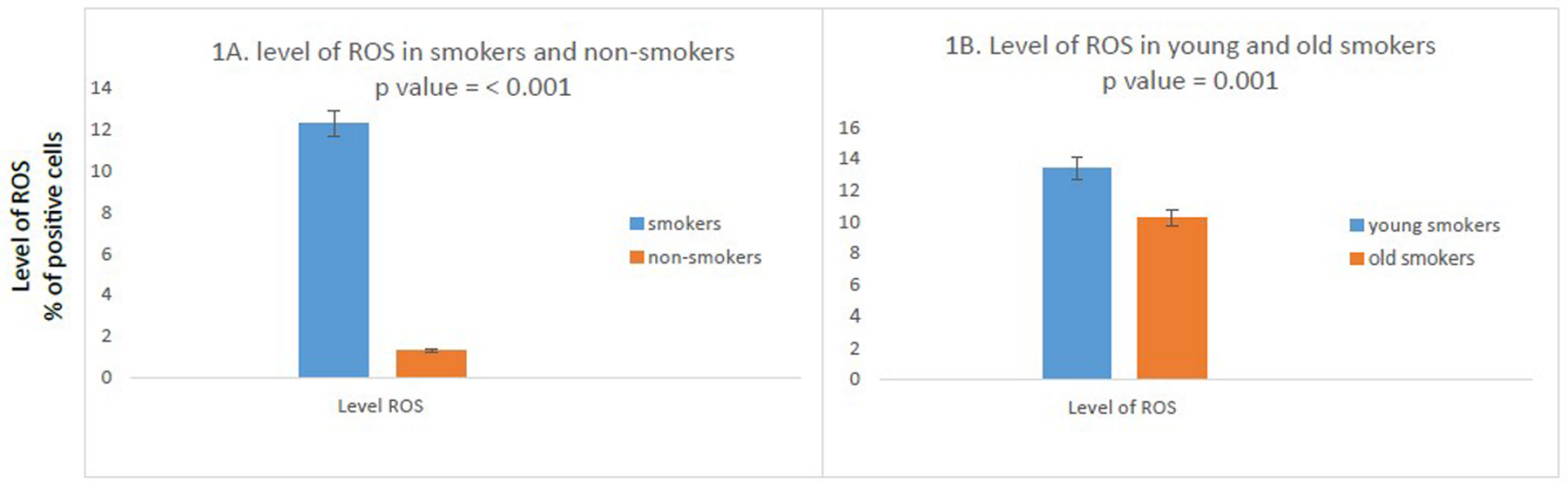
Comparison of ROS in study participants. (*N* = 100). **(A)** High percentage of ROS positive cells in smokers as compared to non-smokers was found. (*n* = 100); smokers *n* = 50; non-smokers *n* = 50. **(B)** Compared to old smokers, young smokers had a higher number of ROS positive cells. (*N* = 50; young smokers *n* = 25; old smokers *n* = 25). The independent T test was used to compare the mean percent positive cells in study participants. *P* value <0.05 was considered as significant as 95% CI.

We found a significant difference in level of ROS observed between smokers and non-smokers. ROS were significantly higher in smokers compared to non-smokers (percentage positivity 12.3260 vs. 1.3280; *p* < 0.001) (Figure 1A).

### Comparison of ROS between young smokers and old smokers

We further compared ROS between young and old smokers, of note, we found the levels of ROS were significantly increased in young smokers as compared to old smokers (percentage positivity 13.396 vs. 10.256; *p* = 0.001) (Figure 1B).

### Comparison of the expression level of CYR61 gene between smokers and non-smokers

**Figure 2:**
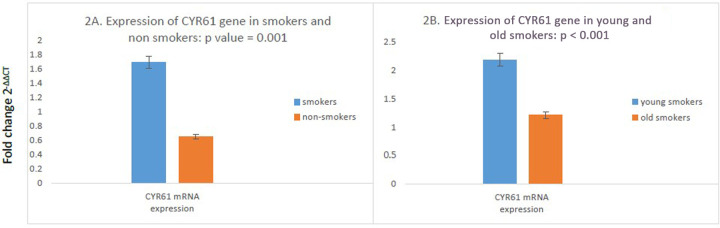
Comparison of mRNA levels of CYR61 in study participants. (*N* = 100). **(A)** High mRNA expression of CYR61 cells in smokers as compared to non-smokers was found. (*n* = 100); smokers *n* = 50; non-smokers *n* = 50. **(B)** Compared to old smokers, young smokers had an increased mRNA expression of CYR61 gene. (*N* = 50; young smokers *n* = 25; old smokers *n* = 25). The independent T test was used to compare the mRNA expressions in study participants. *P* value <0.05 was considered as significant as 95% CI.

A significant difference in CYR61 gene expression was observed between the smokers and non-smokers. It was up regulated in smokers as compared to non-smokers (mean fold change 1.6922 vs. 0.6536; *p* = 0.001) (Figure 2A).

### Comparison of expression level of CYR61 gene between young and old smokers

Moreover, there was a remarkable difference in CYR61 gene expression between young smokers and old smokers. Young smokers were found to have higher expression of CYR61 gene as compared to old smokers. (fold increase 2.1812 vs. 1.2032; *p* = 0.0001) (Figure 2B).

## Discussion

This study demonstrated a significant difference of ROS and CYR61 gene expression between smokers and nonsmokers. Levels of ROS were remarkably higher in smokers, and they exhibited overexpression of CYR61 gene as compared to non-smokers. Smoking cigarettes is an unhealthy practice that impacts human health. Huge amounts of ROS quickly react with numerous molecules within the cell, which affects the cellular physiological processes which in turn increases the risk of age-related diseases like diabetes, cardiovascular disorders, and cancers (14). However, there is still lack of research on how each component in CS can affect multiple human organs, tissues or cells (15).

CS impacts multiple tissues and cells, but limited data is available on oral mucosal cells. A study by Samanta et al. demonstrate that CS exposure produces oxidative damage, not only in lung tissue but also in muscle tissue, having an additional effect on respiratory muscle (16). Another study reveals higher impact of Cigarette Smoke Exposure on Organotypic Bronchial Epithelial Tissue Cultures with production of high ROS (16). Our study also reports the high amount of ROS in smokers and as compared to non-smokers in oral mucosal cells. Over production of ROS causes radical and oxidant-mediated modification micro and macro molecules of the cell which leads to alter the cell physiology including DNA damage, cancers and cell aging (17).

Many studies have shown transcriptome of smokers has altered gene expression and that these alterations are reproducible in different series of smokers (18). Many of those gene families are age-related genes. CS is correlated with up-regulation of the expression of CYR61 gene and DNA damage in buccal epithelial cells of smokers (19). Hyung-Geun et al. reported epithelial cell death and tissue loss in response to prolonged smoking both *in vivo* and *in vitro* (20). Another study revealed CYR61 gene was overexpressed in smokers compared to non- smokers, which was also associated with adipogenesis and inflammation in bronchial epithelial cells (21).

Of note, we compared ROS and cellular aging differences between young and old smokers. Levels of ROS were significantly higher in young smokers compared to old smokers. To the best of our knowledge, this is the first study which has reported the comparison between two smoker groups of different ages for ROS and cellular aging.

Currently the genetic aspects are unable to comprehensively explain the risk and prognosis of cigarette smoking related diseases. Evidence supports the significance of epigenetic alterations due to CS in the start and progression of diseases. Our results are like previous studies which were performed in animal models and humans.

## Conclusion

We found significantly increased ROS and cellular aging in oral mucosal cells of smokers especially in young smokers. This is an alarming situation and might be the leading cause of oral pathologies and other smoking related diseases at a young age.

## Data Availability

The original contributions presented in the study are included in the article/supplementary material, further inquiries can be directed to the corresponding author.
