# Supplementary material for: Higher Reactive Oxygen Species and cellular aging in oral mucosal cells of young smokers: a comparative analytical study
Source: Front Oral Health. 2025 Feb 14;6:1372494. doi: 10.3389/froh.2025.1372494 (PMC11868274; doi:10.3389/froh.2025.1372494)
Supplement: Supplementary file 1 [file Datasheet1.docx]

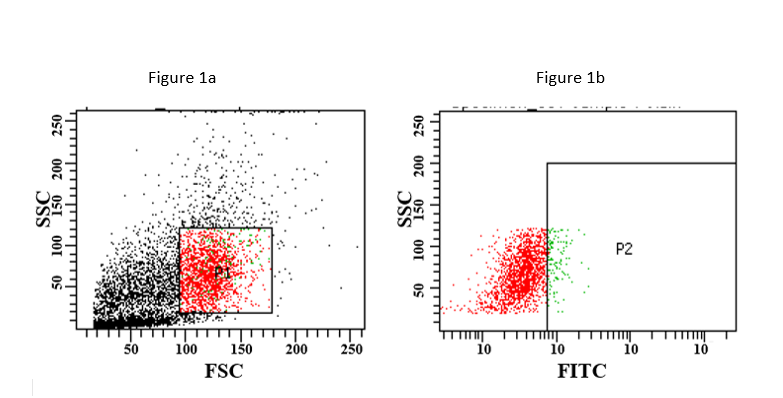


Figure 01: **Figure 1A and B shows DCFDA Stained sample of smoker**


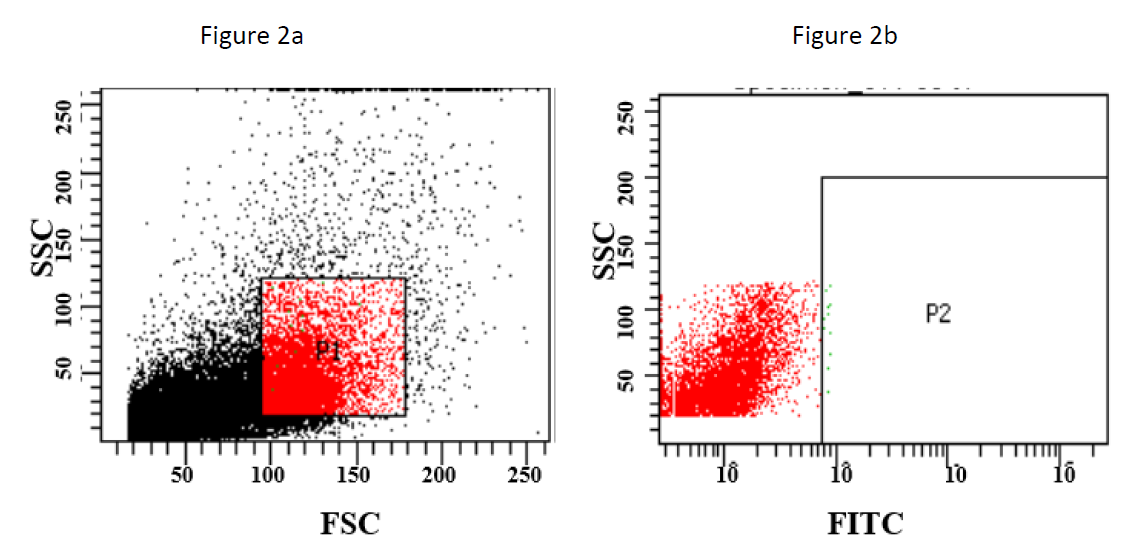


**Figure 2: Figure 2A and B shows DCFDA Stained sample of non-smoker**
